# Antimicrobial Resistance and Cytotoxicity of *Citrobacter* spp. in Maanshan Anhui Province, China

**DOI:** 10.3389/fmicb.2017.01357

**Published:** 2017-07-20

**Authors:** Liyun Liu, Ruiting Lan, Liqin Liu, Yonglu Wang, Yushi Zhang, Yiting Wang, Jianguo Xu

**Affiliations:** ^1^State Key Laboratory of Infectious Disease Prevention and Control, National Institute for Communicable Disease Control and Prevention, Chinese Center for Disease Control and Prevention Beijing, China; ^2^Collaborative Innovation Center for Diagnosis and Treatment of Infectious Diseases Zhejiang, China; ^3^School of Biotechnology and Biomolecular Sciences, University of New South Wales Sydney, NSW, Australia; ^4^Department of Biological Science and Engineering, School of Chemistry and Biological Engineering, University of Science and Technology Beijing Beijing, China; ^5^Maanshan Center for Disease Control and Prevention Maanshan, China

**Keywords:** *Citrobacter*, Multilocus sequence typing, Multidrug resistance, adhesion, cytotoxicity

## Abstract

**Objectives:** *Citrobacter* spp. especially *Citrobacter freundii*, is frequently causing nosocomial infections, and increasingly becoming multi-drug resistant (MDR). In this study, we aimed to determine the genetic diversity and relationships of *Citrobacter* spp. from diarrheal patients and food sources, their antimicrobial resistance profiles and *in vitro* virulence properties.

**Methods:** Sixty two *Citrobacter* isolates, including 13 *C. freundii*, 41 *C. youngae* and eight *C. braakii* isolates, were obtained from human diarrheal patients and food sources. Multilocus Sequence Typing (MLST) of seven housekeeping genes and antimicrobial susceptibility testing using the broth microdilution method according to CLSI recommendations were carried out. Adhesion and cytotoxicity to HEp-2 cells were performed. PCR and sequencing were used to identify *bla*_CTX−M_, *bla*_SHV_, *bla*_TEM_ and *qnr* genes.

**Results:** The 62 isolates were divided into 53 sequence types (STs) with all STs being novel, displaying high genetic diversity. ST39 was a predominant ST shared by 5 *C. youngae* strains isolated from four foods and a diarrheal patient. All isolates were resistant to cefoxitin, and sensitive to imipenem, meropenem and amikacin. The majority of *Citrobacter* isolates (61.3%) were MDR of three or more antibiotics out of the 22 antibiotics tested. Two *C. freundii* isolates each carried the *bla*_TEM−1_ gene and a variant of *qnrB77*. Three *Citrobacter* isolates each carried *qnrS1* and *aac(6')-Ib-cr* genes. Seven isolates that showed strong cytotoxicity to HEp-2 cells were MDR.

**Conclusions:** *Citrobacter* spp. from human and food sources are diverse with variation in virulence properties and antibiotic resistance profiles. Food may be an important source of *Citrobacter* species in transmission to humans. *C. freundii* and *C. youngae* are potential foodborne pathogens.

## Introduction

*Citrobacter* spp. are commensal inhabitants of the intestinal tract of humans and other animals. They have also been recovered from water, sewage, and soil (Nada et al., 2004; Bae et al., 2010). *Citrobacter* spp. are opportunistic pathogens of humans and have been associated with a range of infections including urinary tract infections (UTIs), gastroenteritis, wound infections, pneumonia, brain abscesses, septicaemia, meningitis, and endocarditis, in particular in neonates and immunocompromised hosts (Doran, 1999). *Citrobacter freundii* is the most common *Citrobacter* species causing infections (Mohanty et al., 2007; Samonis et al., 2009; Bai et al., 2012), *C. youngae* and *C. braakii* are rarely a cause of infections. Some *C. freundii* isolates have acquired virulence traits and caused food poisoning or diarrhea in humans (Bai et al., 2012). The main virulence factors found in diarrhea-associated *C. freundii* are toxins, including Shiga-like toxins, heat stable toxins and a cholera toxin B subunit homolog (Bae et al., 2010). In our previous study, we identified one cytotoxic and aggregative *C. freundii* strain and found strains causing diarrheal infections in humans belonged to four sequence types (STs) (Bai et al., 2012). *C. braakii* has been associated with infections, such as hospital-acquired bacteremia and UTIs, making it an opportunistic pathogen (Arens and Verbist, 1997). It was reported that *C. braakii* caused an acute peritonitis in peritoneal dialysis patients (Bai et al., 2012). Moreover, *C. braakii* has been isolated from raw ground beef samples and pork products (Basra et al., 2015; Kwak et al., 2015).

*Citrobacter* spp. as a bacterial contaminant, has been partly responsible for the cause of food-borne diseases, and often transmitted through food and water (Ifeadike et al., 2012). Accordingly, food-handlers with poor personal hygiene could be potential sources of infections by these microorganisms (Ifeadike et al., 2012; Settanni et al., 2013). *Citrobacter* has been isolated from a range of foods (Tassew et al., 2010; Saba and Gonzalez-Zorn, 2012; Kouame et al., 2013) and food poisoning and diarrhea caused by foods contaminated by *Citrobacter* had been reported (Warner et al., 1991; Tschape et al., 1995; Doulgeraki et al., 2011; Giammanco et al., 2011).

Extended spectrum β-lactamases (ESBLs) producing *Citrobacter* strains have been reported. The prevalence of ESBLs varied among countries and *Citrobacter* spp. with reports of 4.9–20.6%, 0.2–4.6%, and 0.9% of *C. freundii* isolates from Korea, Japan and USA, respectively; and 3.5 and 60.0% of *C. koseri* isolates from USA and Japan, respectively (Park et al., 2005; Moland et al., 2006; Choi et al., 2007). Among *Citrobacter* spp. various CTX_−M_ types, SHV and TEM have been reported worldwide (Kanamori et al., 2011).

Plasmid-mediated quinolone resistance genes including *qnr* and *aac(6*′*)-Ibcr* have been reported in *Citrobacter* spp. (Park et al., 2007; Zhang et al., 2012). The *qnr* and *aac(6*′*)-Ibcr* genes were present in 72.8 and 11.6% of clinical *C. freundii* isolates from China, respectively (Zhang et al., 2012). The prevalence of *qnr* genes was found in 38.4% of *C. freundii* isolates in Korea (Park et al., 2007). Numerous *qnrB* alleles have been detected, which seem to be more common than other *qnr* genes (Jacoby et al., 2014). About 40 *qnrB* variants are located on the chromosome of *Citrobacter* spp. especially *C. freundii* (Liao et al., 2015). Of the clinical *C. freundii* isolates with the *qnr* gene, 63.1% carried *qnrB* (Bae et al., 2010).

In this study, we analyzed the genetic diversity by Multilocus Sequence Typing (MLST) and antimicrobial resistance profiles of *Citrobacter* isolates from diarrheal patients, food and food-handlers in Maanshan Anhui Province, China, investigated the prevalence of *bla*_CTX−M_, *bla*_SHV_, *bla*_TEM_ and *qnr* genes and determined the adhesion and cytotoxicity to HEp-2 cells of the isolates.

## Materials and methods

### Ethics statement

This study was reviewed and approved by the ethics committee of National Institute for Communicable Disease Control and Prevention, China CDC. Human fecal pecimens were acquired with the written informed consent of the diarrheal patients and food-handlers with the approval of the ethics committee of National Institute for Communicable Disease Control and Prevention, according to the medical research regulations of Ministry of Health (permit number 2007-17-3).

### *Citrobacter* isolates

Sixty two *Citrobacter* isolates, including 13 *C. freundii*, eight *C. braakii* and 41 *C. youngae* isolates were obtained from patients and food samples from 2007 to 2011 in Maanshan Anhui Province, China. Among these 62 isolates, 18 *C. youngae* and two *C. freundii* isolates were obtained from diarrheal patients. The diarrheal patients harbored no other known enteric bacterial pathogens. Viral causes were not investigated. 42 isolates, including 23 *C. youngae*, 11 *C. freundii* and eight *C. braakii* were isolated from foods (including chicken, pork, fish and vegetables) and food-handlers (Table 1). The identity of each isolate was determined using API 20E test strips (bioMérieux, La Balme les Grottes, France) at the time of isolation, and they were stored as glycerol stocks at −80°C. Bacteria were grown in Luria-Bertani (LB) broth or on LB and Mueller–Hinton agar plates (pH 7.4) at 37°C.

**Table 1:** *Citrobacter* isolates used in this study and their characteristics.

**Isolates**	**Species**	**STs**	**Year**	**Source**	**Adhesion**	**LDH**
AH2007001	*C. youngae*	25	2007	Diarrheal patient	^**^	22.4 ± 1.1
AH2007002	*C. youngae*	25	2007	Diarrheal patient	^**^	9.8 ± 0.7
AH2007003	*C.youngae*	26	2007	Diarrheal patient	^**^	5.9 ± 0.1
AH2007004	*C. youngae*	27	2007	Diarrheal patient	^**^	37.1 ± 2.6
AH2007006	*C. youngae*	28	2007	Diarrheal patient	^***^	24.1 ± 0.5
AH2007007	*C. youngae*	28	2007	Diarrheal patient	^**^	8.1 ± 0.5
AH2007008	*C. youngae*	29	2007	Diarrheal patient	^**^	22.3 ± 1.8
AH2007009	*C. youngae*	30	2007	Diarrheal patient	^**^	3.7 ± 1.2
AH2007010	*C. youngae*	31	2007	Diarrheal patient	^**^	0.1 ± 1.3
AH2007013	*C. youngae*	32	2007	Diarrheal patient	^*^	3.2 ± 0.8
AH2007014	*C. freundii*	33	2007	Diarrheal patient	^*^	5.3±.08
AH2007015	*C. youngae*	34	2007	Diarrheal patient	^**^	18.7 ± 6.4
AH2007016	*C. youngae*	35	2007	Diarrheal patient	^*^	1.3 ± 0.5
AH2007018	*C. freundii*	36	2007	Diarrheal patient	^*^	16.7 ± 4.1
AH2007019	*C. youngae*	37	2007	Diarrheal patient	^*^	4.2 ± 4.2
AH2007021	*C. youngae*	38	2007	Diarrheal patient	^*^	11.5 ± 1.3
AH2007022	*C. youngae*	39	2007	Diarrheal patient	^**^	21.4 ± 5.8
AH2007023	*C. youngae*	40	2007	Food-handler	^**^	6.6 ± 0.4
AH2007024	*C. youngae*	39	2007	Tofu skin	^**^	11.9 ± 0.2
AH2007025	*C. youngae*	39	2007	Pig's ear	^*^	0.1 ± 0.5
AH2007026	*C. youngae*	41	2007	Food-handler	^***^	36.5 ± 2.4
AH2008001	*C. youngae*	39	2008	Beef	^*^	3.3 ± 0.4
AH2008002	*C. youngae*	39	2008	Egg	^*^	19.3 ± 1.3
AH2008004	*C. freundii*	42	2008	Carp meat	^**^	30 ± 2.3
AH2008005	*C. freundii*	43	2008	Duck leg	—	14.9 ± 7.8
AH2008006	*C. freundii*	44	2008	Carp meat	^**^	0.1 ± 0.4
AH2008007	*C. freundii*	45	2008	Flower silver carp	^*^	0.7 ± 0.4
AH2008008	*C. freundii*	46	2008	Duck leg	^**^	11.0 ± 2.0
AH2008009	*C. freundii*	47	2008	Pigeon meat	^***^	20.2 ± 5.4
AH2008010	*C. youngae*	48	2008	Carp meat	^**^	29.2 ± 2.3
AH2008011	*C. youngae*	49	2008	Chicken breast	^*^	5.7 ± 0.2
AH2008012	*C. youngae*	50	2008	Anchovy	^**^	9.2 ± 0.2
AH2008014	*C. braakii*	51	2008	Duck neck	—	4.4 ± 1.8
AH2008015	*C. braakii*	52	2008	Food-handler	±	11.0 ± 4.6
AH2008016	*C. youngae*	53	2008	Food-handler	±	4.4 ± 0.7
AH2009001	*C. freundii*	54	2009	Pork liver	±	2.2 ± 1.2
AH2009002	*C. braakii*	55	2009	Carp meat	^*^	1.7 ± 0.1
AH2009003	*C. youngae*	56	2009	Carp meat	^**^	14 ± 1.6
AH2009004	*C. youngae*	57	2009	Pork	^**^	5.2 ± 0.2
AH2009006	*C. braakii*	58	2009	Meat	^**^	0.5 ± 0.1
AH2009007	*C. youngae*	59	2009	Meat	^**^	19.8 ± 3.9
AH2009008	*C. youngae*	60	2009	Catfish	^*^	0.1 ± 2.4
AH2009009	*C. youngae*	59	2009	Chicken thigh	^*^	13.5 ± 2.3
AH2009010	*C. youngae*	71	2009	Pork	^***^	60.4 ± 2.7
AH2009011	*C. youngae*	72	2009	Meat	^**^	29.4 ± 3.8
AH2009012	*C. youngae*	59	2009	Pomfret	±	4.8 ± 0.8
AH2009013	*C. youngae*	73	2009	Diarrheal patient	±	13.2 ± 0.7
AH2009014	*C. youngae*	74	2009	Diarrheal patient	±	1.4 ± 0.7
AH2009015	*C. youngae*	75	2009	Diarrheal patient	^*^	15.2 ± 2.8
AH2009016	*C. youngae*	76	2009	Fish heads	—	0.1 ± 0.1
AH2009017	*C. youngae*	77	2009	Yellow-fin tuna	^*^	2.2 ± 1.1
AH2009018	*C. braakii*	78	2009	Pork	—	4 ± 0.5
AH2010001	*C. braakii*	79	2010	Carp meat	—	0.1 ± 0.5
AH2010002	*C. youngae*	80	2010	Pork	^**^	0.1 ± 1.3
AH2011001	*C. braakii*	81	2011	Carp meat	—	0.7 ± 0.1
AH2011002	*C. braakii*	82	2011	Carp meat	—	0.1 ± 0.3
AH2011005	*C. youngae*	83	2011	Water	^*^	6.4 ± 1.9
AH2011006	*C. freundii*	84	2011	Flat fish	^**^	3.5 ± 0.4
AH2011007	*C. freundii*	85	2011	Catfish	±	0.2 ± 0.2
AH2011008	*C. freundii*	86	2011	Tofu	^*^	0.1 ± 0.1
AH2011009	*C. freundii*	86	2011	Spiced duck	^**^	4.2 ± 1.3
AH2011010	*C. youngae*	87	2011	Snake melon salad	^*^	15.7 ± 0.1

***, **, * *correspond to adhesion index of >50, >1, and <50 and <1 respectively. ± means ambivalent or no adhesion, −means no adhesion*.

### Multilocus sequence typing and phylogenetic analysis

The *Citrobacter* MLST scheme (http://pubmlst.org/cfreundii/) was used. The seven housekeeping genes for MLST were *aspC, clpX, fadD, mdh, arcA, dnaG* and *lysP*, and the MLST primers were as previously described (Bai et al., 2012) and synthesized by Shanghai Sangon Biological Engineering Technology and Services (Shanghai, China). PCR products were verified on 1% agarose gels and purified. DNA sequence was determined using Sanger sequencing in both directions (Shanghai Sangon Biological Engineering Technology and Services, China). Sequences were analyzed using SeqMan 7.0 software.

### Antimicrobial susceptibility testing

Antimicrobial susceptibility testing was carried out using the broth microdilution method according to CLSI recommendations. Minimum inhibitory concentration (MIC) results were interpreted according to the European Committee on Antimicrobial Susceptibility Testing (EUCAST) guidelines. The antibiotics were serially diluted 2-fold in 50 μL of cation-adjusted Mueller-Hinton broth. The bacterial suspension was prepared from actively growing bacteria in 5 mL of cation-adjusted Mueller-Hinton broth, and diluted to a bacterial cell density of 10^6^ colony forming units (CFU)/mL. Five microliter of bacterial suspension was then added to wells containing 100 μL of serially diluted antimicrobial agents to yield a final inoculum of approximately 5 × 10^4^ CFU/mL. The MICs were read after overnight incubation (18–24 h) at 35°C. Quality control for MICs was performed using the reference *E. coli* ATCC 25922.

### PCR amplification and sequencing

All the isolates were screened for the following genes, *qnrA, qnrB, qnrS, qnrC, qnrD, aac(6*′*)-Ib-cr, qepA, bla*_CTX−M_, *bla*_SHV_, and *bla*_TEM_ by PCR using primers listed in Table S1. Primers of *qnrA, qnrB, qnrS, qnrC, qnrD, aac(6*′*)-Ib-cr*, and *qepA* were from Shao *et al* (Shao et al., 2011), primers for screening *bla*_CTX−M_, *bla*_SHV_ and *bla*_TEM_ genes were from Zhang et al. (2011). All primers were synthesized by Shanghai Sangon Biological Engineering Technology and Services (Shanghai, China). Positive PCR products were confirmed by sequencing.

### *In vitro* adhesion and cytotoxicity assays

*In vitro* adhesion to host cells was performed using the human epidermoid carcinoma cell line HEp-2 (CCC0068; Beijing Union Medical College cell resource center), as previously described (Bai et al., 2012). An adhesion index (<1; >1 and <50; >50) describing the mean number of bacteria per HEp-2 after examination of 10 visual fields was determined (Bai et al., 2012). Infections were repeated three times in duplicate.

The lactate dehydrogenase (LDH) released by the HEp-2 cells was determined using the Cytotox96 kit (Promega) according to the manufacturer's instructions. The relative amount of cytotoxicity was expressed as follows: (experimental release–spontaneous release)/(maximum release–spontaneous release)X100, where the spontaneous release was the amount of LDH activity in the supernatant of uninfected cells and the maximum release was that when cells were lysed with the lysis buffer provided by the manufacturer. All experiments were performed two times in duplicate (Bai et al., 2012).

## Results

### Multilocus sequence typing of *Citrobacter* isolates

The 62 *Citrobacter* isolates including 13 *C. freundii*, 41 *C. younga*e and eight *C. Braakii* isolates were divided into 53 STs by MLST (Table 1). The 41 *C. younga*e isolates were divided into 32 STs, 13 *C. freundii* isolates into 12 STs and eight *C. Braakii* isolates into 8 STs. Four STs (ST25, ST28, ST39, and ST59), all belonging to *C. youngae*, contained multiple isolates from two to five isolates. ST25 and ST28 each contained two isolates from diarrheal patients. ST39 contained five isolates with one from a diarrheal patient and four from foods. All three ST59 isolates were from foods.

**Figure 1:**
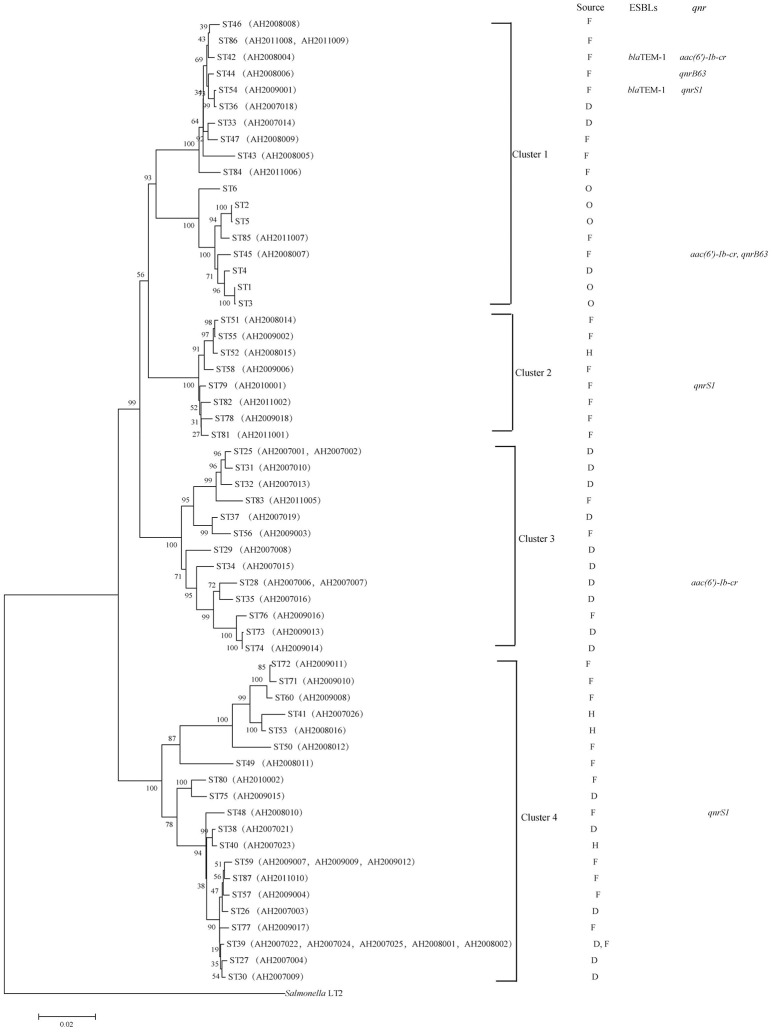
Phylogenetic relationships as determined by MLST data. The presence of ESBLs and *qnr* genes among the *Citrobacter* isolates is shown on the right. The tree was constructed using neighbor joining algorithm. For each ST, F, D, H, and O indicate isolates from foods, diarrheal patients, food-handlers and animals respectively. Cluster divisions are marked. Numbers on near the nodes are bootstrap values from 1,000 replicates.

A phylogenetic tree for the 62 isolates and representative isolates for ST1 to ST6 reported previously (Bai et al., 2012) was constructed using the neighbor-joining algorithm based on the concatenated sequences of the seven housekeeping genes (Figure 1). *Salmonella LT2* was used as an outgroup. The tree could be divided into four clusters with robust bootstrap support of the major divisions. Cluster 1 is comprised of all *C. freundii* isolates; cluster 2 is comprised of all *C. braakii* isolates; and Cluster 3 and cluster 4 are comprised of all *C. youngae* isolates. It is interesting to note that clusters 3 and 4 are not grouped together. Rather, cluster 3 is grouped with clusters 1 and 2 with 90% bootstrap support, suggesting that cluster 3 should be a separate species from cluster 4. However, more isolates are needed to get a better understanding of the diversity of these 3 species and their relationships.

### Antimicrobial susceptibility

The 62 *Citrobacter* isolates were tested for susceptibility to 22 antibiotics using the broth microdilution method according to CLSI recommendations (Table 2). All were resistant to cefoxitin (CFX), and sensitive to imipenem (IMI), meropenem (MEM) and amikacin (AMI). Non-susceptibility to β-lactams ranged from 0% to 100%; non-susceptibility to the three quinolones tested ranged from 12.9% to 27.4%; and non-susceptibility to other antibiotics included aminoglycosides (0–12.9%), phenicols (12.9%), sulfonamides (12.9–25.8%), tetracyclines (25.8%), and macrolide (3.2%) (Table 2).

**Table 2:** Number of isolates non-susceptible to antibiotics by species and source.

**Antibiotic**	***C. freundii* (*n* = 13)**		***C. youngae* (*n* = 41)**		***C.braakii* (*n* = 8)**	
	**D (*n* = 2)**	**F (*n* = 11)**	**D, H (*n* = 20)**	**F (*n* = 21)**	**H (*n* = 1)**	**F (*n* = 7)**
	**Resistant (number)**		**Resistant (number)**		**Resistant (number)**	
**β-LACTAMS**						
Ampicillin	2	7	20	14	1	5
Cefotaxime	0	2	3	2	0	0
Ceftazidime	0	4	4	6	0	3
Cefepime	0	0	2	0	0	0
Cefoxitin	2	11	20	21	1	7
Imipenem	0	0	0	0	0	0
Aztreonam	0	0	1	2	0	0
Meropenem	0	0	0	0	0	0
Ceftiofur Sodium	0	1	3	1	0	0
**QUINOLONES**						
Nalidixicacid	1	6	2	4	0	4
Ciprofloxacin	1	2	0	4	0	1
Levofloxacin	0	3	1	4	0	2
**AMINOGLYCOSIDES**						
Gentamicin	0	1	0	0	0	0
Amikacin	0	0	0	0	0	0
Streptomycin	0	3	0	4	0	1
Kanamycin	0	1	1	4	0	0
**TETRACYCLINES**						
Tetracycline	0	5	3	5	0	3
Doxycycline	0	4	4	5	0	3
**PHENICOLS**						
Chloramphenicol	0	3	0	4	0	1
**SULFONAMIDES**						
Trimethoprim/Sulfamethoxazole	0	4	5	5	1	1
Sulfafurazole	0	3	0	3	0	2
**MACROLIDE**						
Azithromycin	0	1	1	0	0	0

*D, diarrheal patients; H, food-handlers; F, foods*.

Among the 62 *Citrobacter* isolates tested for MIC to 22 antibiotics, six *C. youngae*, seven *C. freundii*, and four *C. braakii* isolates were highly resistant to NAL, with MICs > 128 μg/mL and were multidrug resistant, with resistance to ≥3 antibiotics. Among 17 NAL resistant isolates, 14 isolates were from food and three were from diarrheal patients. These isolates belonged to different phylogenetic clusters, seven in cluster 1, four in cluster 2, one in cluster 3 and five in cluster 4. Three *C. youngae* isolates (one in cluster 3 and two in cluster 4) had a CTX MIC of > 16 μg/mL, and were multidrug resistant, with resistance to ≥8 antibiotics and were not closely related by MLST (Figure 1 and Table 3). There are six isolates (three *C. youngae*, two *C. freundii*, and one *C. braakii*) that had high MIC to CHL (>32 μg/mL), STR (>32 μg/mL), Sul (>512 μg/mL), TET (>32 μg/mL) and SXT (>8/152 μg/mL) (Table 3). The three highly multidrug resistant *C. youngae* isolates were separated on the tree but all in cluster 4 (Figure 1 and Table 3).

**Table 3:** MIC values (μg/mL) of antimicrobial Resistance in 62 *Citrobacter* Isolates.

**Isolates**	**Source**	**Antibiotics**																
		**AMP**	**AZM**	**FEP**	**CAZ**	**CLP**	**LEV**	**SXT**	**CTX**	**TIO**	**NAL**	**CHL**	**STR**	**SUL**	**TET**	**AMZ**	**KAN**	**DOX**
AH2007001	D				16										32			16
AH2007002	D	32						>8/152										
AH2007003	D	32		>32														
AH2007004	D	32							4	8								
AH2007006	D	32		>32					>16	>32	>128				>32	>64	>64	8
AH2007007	D	32						>8/152										
AH2007008	D	32			16													
AH2007009	D	32	>32		>32				>16	>32					>32			>16
AH2007010	D	32																
AH2007013	D	32																
AH2007014	D	32				8					>128							
AH2007015	D	32						>8/152										
AH2007016	D	32						>8/152										
AH2007018	D	32																
AH2007019	D	32																
AH2007021	D	64					16				>128							
AH2007022	D	64																
AH2007023	F	32																
AH2007024	F	32			16													
AH2007025	F	64	32		>32				>16	>32					32			>16
AH2007026	H	>128			16													8
AH2008001	F	32																
AH2008002	F	32			16				4									
AH2008004	F	>128				>32	>16	>8/152	4		>128	>32	>32	>512	>32	64	>64	>16
AH2008005	F	32			16						>128				>32			>16
AH2008006	F	32					16				>128							
AH2008007	F	32			16						>128				>32			8
AH2008008	F							>8/152										
AH2008009	F																	
AH2008010	F	>128				8	>16	>8/152			>128	>32	>32	>512	>32			16
AH2008011	F	32																
AH2008012	F																	
AH2008014	F	32																
AH2008015	H	32						>8/152										
AH2008016	H	32																
AH2009001	F	32				8	>16	>8/152	8	8	>128	>32	>32	>512	>32			16
AH2009002	F	32			16									>512	>32			16
AH2009003	F	>128	32														64	
AH2009004	F	32																
AH2009006	F																	
AH2009007	F	32			16													
AH2009008	F							>8/152										
AH2009009	F																	
AH2009010	F					8	16	>8/152			>128	>32	>32		>32		>64	>16
AH2009011	F	32				4	16	>8/152			>128	>32	>32	>512	>32		>64	>16
AH2009012	F																	
AH2009013	D	64																
AH2009014	D	32						>8/152										
AH2009015	D	32																
AH2009016	F	32																
AH2009017	F					4	16	>8/152			>128	>32	>32	>512	>32		>64	16
AH2009018	F	32			16						>128				32			16
AH2010001	F	32				8	16	>8/152			>128	>32	>32	>512	>32			16
AH2010002	F	32			16													
AH2011001	F	32					16				>128							
AH2011002	F	32			16						>128							
AH2011005	F				16													
AH2011006	F	32																
AH2011007	F				16						>128	>32	>32	>512	>32			
AH2011008	F	32			16													
AH2011009	F							>8/152										
AH2011010	F	32																

*D, diarrheal patients; H, food-handlers; F, foods; MIC, minimum inhibitory concentration; AMP:ampicillin;CTX, cefotaxime; CAZ, ceftazidime; FEP, cefepime; TIO, ceftiofur Sodium; AZM, aztreonam; NAL, nalidixicacid; CLP, ciprofloxacin; LEV, levofloxacin;CHL, chloramphenicol; STR, streptomycin; SUL, sulfafurazole; TET, tetracycline; SXT, trimethoprim/Sulfamethoxazole; AMZ, azithromycin; KAN, kanamycin; DOX, doxycycline*.

### Detection of *bla*_CTX−M_, *bla*_SHV_, *bla*_TEM_, and *qnr* genes by PCR

Two *C. freundii* isolates (AH2008004 and AH2009001) were found to harbor a *bla*_TEM−1_ gene by PCR and sequencing, both of which were resistant to AMP, CLP, LEV, SXT, CTX, NAL, CHL, STR, SUL, TET, CFX, and DOX. However, the two *bla*_TEM−1_ positive isolates belonged to two different STs with AH2008004 belonging to ST42 and AH2009001 belonging to ST54 (Figure 1 and Table 3).

Three isolates were positive for *qnrS1* including one *C. youngae* (AH2008010), one *C. freundii* (AH2009001) and one *C. braakii* isolate (AH2010001). One *C. youngae* (AH2007006) and two C. *freundii* isolates (AH2008004 and AH2008007) were found to harbor an *aac(6*′*)-Ib-cr* gene. These two *C. freundii* isolates belonged to two different STs (Figure 1 and Table 1).

Two *C. freundii* isolates (AH2008006 and AH2008007) were found to harbor a *qnrB* gene. This *qnrB* allele has two in-phase ATG start codons. Wang et al. reported that two in-phase ATG start codons are present in many *qnrB* alleles (*qnrB1, qnrB3, and qnrB5*). However, in *qnrB2* and *qnrB4*, the first ATG is out of phase with the remainder of the reading frame, the translation may be initiated at the second ATG codon (Wang et al., 2009). If sequence analysis from the ATG at position 37 (the second ATG codon), our *qnrB* allele has an identical *qnrB* sequence as *qnrB77*. But *qnrB77* (GenBank accession no. KM985470.1) did not contain this 36 bp region. The 36 bp in our *qnrB* contained a LexA binding site (Wang et al., 2009). Therefore, we suggest that our *qnrB* allele is a variant of *qnrB77*.

These two *qnrB* positive isolates AH2008006 and AH2008007 belonged to two different STs, ST44 and ST45, respectively, suggesting that these isolates were epidemiologically unrelated (Figure 1).

### HEp-2 cell adherence of *Citrobacter* isolates

Adhesion is an essential virulence property of bacterial pathogens. *In vitro* assays have been widely used to assess this property (Mange et al., 2006). We tested the 62 isolates for adhesion to HEp-2 cells and categorized the extent of adhesion using the adhesive index (Mange et al., 2006) (Table 1). Four isolates (including three *C. youngae* and one *C. freundii*) showed the strongest adhesion, with adhesion indexes >50. Twenty-five isolates showed intermediate adhesion, with an adhesion index between 1 and 50. Nineteen isolates showed little adhesion, with an adhesion index of <1. The remaining isolates showed ambivalent adhesion or no adhesion.

The adhesion rate was lower for *C. braakii* (25%) than *C*. *youngae* (88%) and *C. freundii* (77%). No difference was evident (*P* > 0.05) when adhesion behavior was compared in view of the source (human and food) of the *Citrobacter* isolates.

### HEp-2 cell cytotoxicity of *Citrobacter* isolates

**Figure 2:**
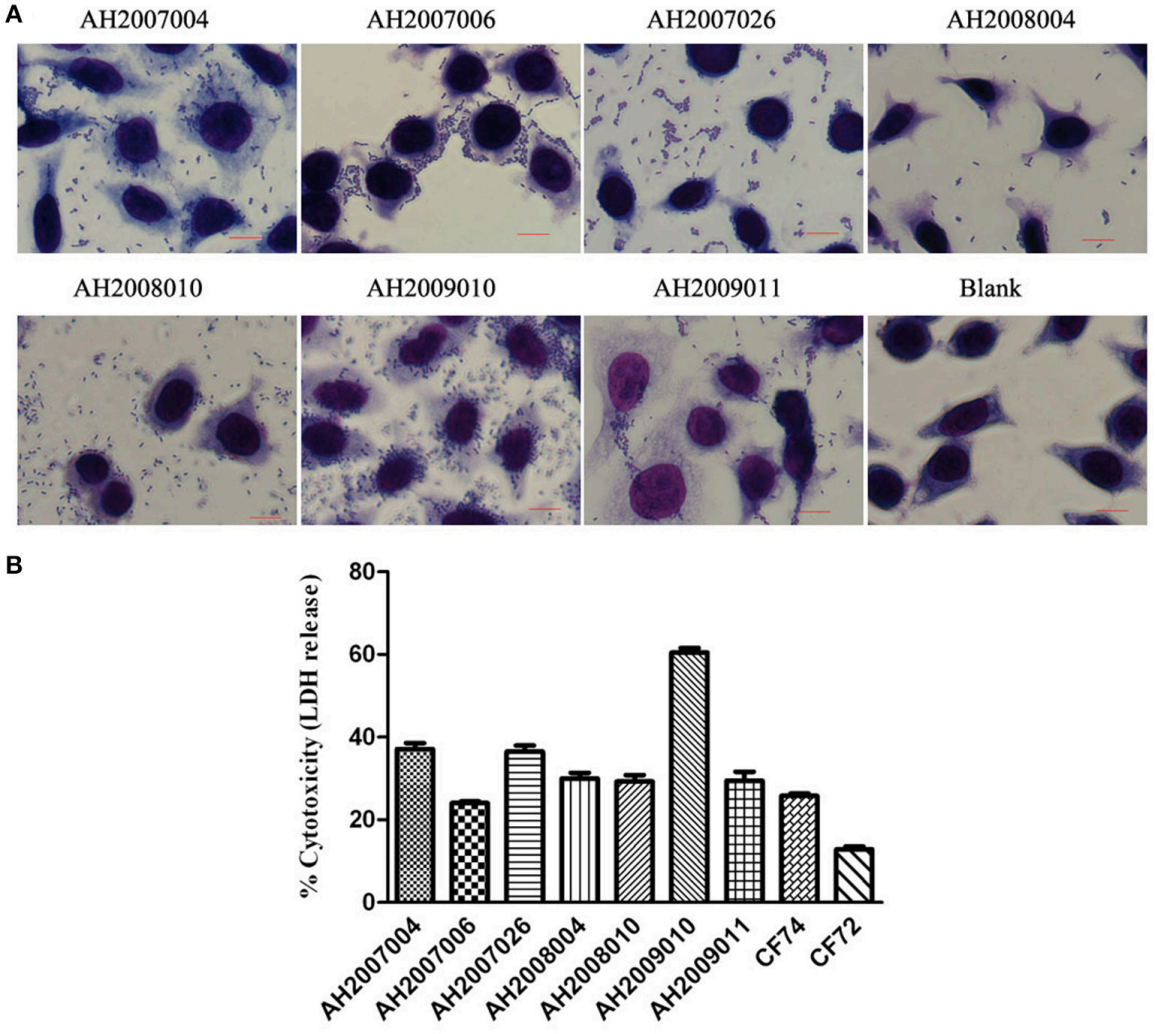
HEp-2 cell adhesion and cytotoxicity of *Citrobacter* isolates. **(A)** Light micrographs show the adherence patterns displayed by seven cytotoxic *Citrobacter* isolates. Blank as negative control. Bar: 10 μm. **(B)** Cytotoxicity was based the LDH released from HEp-2 cells after exposure to cytotoxic *Citrobacter* isolatesat 8 h. CF72 and CF74 were control strains.

The 62 *Citrobacter* isolates were tested for Cytotoxicity to cultured HEp-2 cells by measuring the amount of lactate dehydrogenase (LDH) released by HEp-2 cells. We tested all isolates at 8 h. The released LDH levels ranged from 0.1–60.0% (Table 3). *C. freundii* strain CF74 were used as a positive control of cytotoxicity and *C. freundii* strain CF72 was used as a negative control (Bai et al., 2012). The levels of LDH released by CF74 and CF72 were 25.7 and 12.8% respectively. Seven isolates (including five *C. youngae* and two *C. freundii* isolates) released LDH more than 24%, showing strong cytotoxicity (Table 1). Among these seven isolates, three isolates showed strongest adherence; four isolates showed intermediate adhesion (Figure 2). Another seven isolates (including six *C. youngae* and one *C. freundii* isolates) released LDH from 18.7 to 22.4% and are considered intermediate cytotoxic. The remaining 48 isolates showed LDH release <16.7% and are likely to be non-cytotoxic.

Seven strongly cytotoxic isolates were multidrug resistant, with resistance to ≥3 antibiotics (Tables 1, 3). Four isolates (AH2008004, AH2008010, AH2009010, and AH2009011) showed multi-drug resistant (MDR) to nine antibiotics (CFX, NAL, CLP, LEV, CHL, STR, TET, SXT, and DOX). Moreover, AH2007006 harbored an *aac(6*′*)-Ib-cr* gene, AH2008004 harbored a *bla*_TEM−1_ gene and an *aac(6*′*)-Ib-cr* gene, and AH2008010 harbored a *qnrS1* gene.

Four intermediate cytotoxic isolates (including AH2007001, AH2007008, AH2008002 and AH2009007) were resistant to AMP, CAZ and CFX (Tables 1, 3).

## Discussion

*Citrobacter* spp. especially *C. freundii*, is recognized as an emerging opportunistic pathogen and is known to cause a variety of infections (UTIs, wound infections, gastrointestinal infections, septicemia, meningitis), especially in immunocompromised patients and in hospital settings (Joaquin et al., 1991; Brenner et al., 1993; Gupta et al., 2003; Samonis et al., 2009; Ranjan and Ranjan, 2013; Leski et al., 2016a). This emergence has coincided with the finding that *C. freundii* is often resistant to multiple classes of antibiotics, suggesting that both clinical and environmental strains may be a reservoir of antimicrobial resistance determinants (Pepperell et al., 2002; Nada et al., 2004; Yim et al., 2013; Feng et al., 2015; Leski et al., 2016a; Sheppard et al., 2016). A recent survey of outpatients in Bo, Sierra Leone, revealed that a surprisingly high number of *C. freundii* isolates from UTIs were highly MDR (Leski et al., 2016b). In this study, we surveyed *Citrobacter* species from diarrheal patients and foods to provide a better understanding of their genetic diversity, antibiotic resistance profile, virulence properties and their potential as foodborne pathogens.

The worldwide prevalence of ESBLs in *Citrobacter* spp. was reported to be 0.5–36% (Ali et al., 2004; Fernandes et al., 2014; Praharaj et al., 2016). In India, 80.9% of *Citrobacter* isolates from hospitalized patients were ESBL producers (Praharaj et al., 2016). In this study, we did not test for ESBL phenotype but screened by PCR for *Bla*_CTX−M_, *bla*_TEM_ and *bla*_SHV_ genes. We found that a very low percentage of our isolates were *bla*_TEM−1_ positive (3.2%) and none carried *Bla*_CTX−M_ and *bla*_SHV_. In contrast to a study in India, Shahid (Shahid, 2010) found that *Bla*_CTX−M_, *bla*_TEM_ and *bla*_SHV_ were found in 67.5%, 40%, and 25% of *Citrobacter* isolates from human clinical infections, respectively. However, most of our isolates were from food sources.

The prevalence of *qnr* and *aac(6*′*)-Ib-cr* genes varied. A Korean study showed that 38.4% of *C. freundii* isolates harbored *qnr* determinants (Park et al., 2007). A study from China showed prevalence of *qnr* and *aac(6*′*)-Ib-cr* genes at 63.3% and 26.7% in *C. freundii* isolates, respectively (Yang et al., 2008), while another Chinese study showed the prevalence of *qnr* and *aac(6*′*)-Ib-cr* in *C. freundii* at 72.8% and 68.9%, respectively (Zhang et al., 2012). The latter study also reported the prevalence of *qnr* and *aac(6*′*)-Ib-cr* in *C. braakii* at 42.9% and 42.9%, respectively (Zhang et al., 2012). We found much lower prevalence of *qnr* and *aac(6*′*)-Ib-cr* genes at 23.1% and 15.4% in *C. freundii* isolates; 2.4% and 2.4% in *C. youngae* isolates, and 12.5% and 0% in *C. braakii* isolates, respectively.

*QnrB* is the most common of the five *qnr* families and has the greatest number of allelic variants (Jacoby et al., 2011). We found a variant of *qnrB77* in two *C. freundii* isolates. The variant contained a 36 bp sequence upstream of the *qnrB77* start codon with an in-phase ATG codon at the beginning and a LexA binding site within the sequence, similar to several other *qnrB* alleles. The study by Wang et al. showed that the LexA binding site renders the *qnrB* under SOS control leading to its higher expression in response to ciprofloxacin or mitomycin C treatment (Wang et al., 2009). However, it should be noted that the *qnrB77* first reported has no upstream sequence available in the GenBank entry and therefore it cannot be ascertained whether the sequence was absent or not reported.

*QnrB*-carrying *C. freundii* isolates do not always show high level of quinolone resistance (Zhang et al., 2012). However, our two *qnrB*-carrying *C. freundii* had a high MIC for NAL (>128 μg/mL). *C. freundii* carrying *qnrS* and *aac(6*′*)-Ib-cr* have been shown to have a higher MIC for quinolones (Zhang et al., 2012). Our results are consistent with this observation. One *aac(6*′*)-Ib-cr*-carrying *C. freundii* and three *qnrS1*-carrying *Citrobacter* isolates had high MIC of three quinolones (NAL, >128 μg/mL; CLP, >32 μg/mL; LEV, >16 μg/mL).

High prevalence of multidrug resistant *Citrobacter* has been reported (Moges et al., 2014; Leski et al., 2016b). Moges et al found that 13 MDR *Citrobacter* spp. were isolated from waste water in hospital and non-hospital environments (Moges et al., 2014). Twenty-two MDR *C. freundii* isolates from outpatient urine samples were resistant to >7 antibiotics out of the 11 tested, and 81.8% of the *C. freundii* isolates produced ESBLs (Leski et al., 2016b). In this study, 61.3% *Citrobacter* isolates were resistant to ≥3 antibiotics out of the 22 tested, and seven MDR isolates were strongly cytotoxic and four were intermediately cytotoxic. Moreover, two of the seven strongly cytotoxic and MDR isolates (from *C. youngae*) were obtained from diarrheal patients. The cytotoxic property of these isolates implies that they may cause more severe disease while the MDR properties limit clinical therapeutic options.

*Citrobacter youngae* is rarely a cause of infections. It has been reported to cause peritonitis (Chen et al., 2013). However, *C. younage* has not been recognized as a diarrheal pathogen. We found that 50% of the isolates showed moderate to strong adhesion and 15% of the isolates also showed strong cytoxicity. Nearly half of the *C. younage* isolates were from diarrheal patients. However, not all human isolates were adhesive or cytotoxic. Three of the six adhesive and cytotoxic isolates were obtained from diarrheal patients, suggesting that such strains are likely to cause diarrheal disease. STs from both human and food isolates were diverse with most STs being only isolated once. However, three STs were isolated more than once. Interestingly one ST was isolated from food as well as from a diarrheal patient. These findings suggest that *C. youngae* is a potential foodborne diarrheal pathogen.

*Citrobacter freundii* is the most common cause of *Citrobacter* infections (Mohanty et al., 2007; Samonis et al., 2009) and has been implicated in gastroenteritis associated outbreaks (Guerrant et al., 1976; Warner et al., 1991; Tschape et al., 1995; Doulgeraki et al., 2011; Giammanco et al., 2011) and foodborne outbreaks (Ifeadike et al., 2012; Settanni et al., 2013). We only obtained two isolates from diarrheal patients. Neither isolate was adhesive and one of them was intermediately cytotoxic, questioning its role in diarrhea in these cases. However, five isolates from foods were adhesive or strongly cytotoxic, suggesting that food isolates serve as a potential foodborne pathogen. The STs from this study were compared with six STs (ST1-ST6) from our previous study and 28 STs from the *Citrobacter* MLST database of global isolates, all STs found in this study were novel STs, showing high diversity of *C. freundii* from different regions and countries.

*Citrobacter braakii* is commonly found in water, soil, food, and the intestinal tracts of animals and humans (Basra et al., 2015). *C. braakii* is an opportunistic pathogen and has been isolated from hospital infections and UTIs (Arens and Verbist, 1997). *C. braakii* can cause acute peritonitis in peritoneal dialysis patients (Chao et al., 2013). All eight *C. Braakii* isolates from this study were isolated from foods. It requires further study to determine whether *C. braakii* contributes to diarrheal disease.

## Conclusion

We analyzed 13 *C. freundii*, 41 *C. youngae*, and eight *C. braakii* isolates from Maanshan Anhui Province, China, isolated from human diarrheal patients and foods for their genetic diversity, antibiotic sensitivity and *in vitro* virulence phenotype. The 62 isolates were divided into 53 STs with all STs being novel, displaying high genetic diversity. Half of the isolates were MDR of three or more antibiotics. The *bla*_TEM−1_ gene was detected in two *C. freundii* isolates, while *qnrS1* and *aac(6*′*)-Ib-cr* genes were detected in three *Citrobacter* isolates, respectively. We found seven isolates that showed strong cytotoxicity to HEp-2 cells, all of which were MDR. We also found a variant of *qnrB77* that contained a LexA site in two *C. freundii* isolates. Our data suggest that food is an important source of *Citrobacter* species in transmission to humans and *C. freundii* and *C. youngae* are potential foodborne pathogens. Further studies are required to determine their public health significance.

## Author contributions

LyL and JX designed the project; YlW carried out the sampling work; YZ carried out the experiments; YtW, LqL, and RL analyzed data; LyL and RL drafted the manuscript. All authors have read and approved the final version of the manuscript.

### Conflict of interest statement

The authors declare that the research was conducted in the absence of any commercial or financial relationships that could be construed as a potential conflict of interest.
