# Supplementary material for: Antimicrobial Resistance and Cytotoxicity of Citrobacter spp. in Maanshan Anhui Province, China
Source: Front Microbiol. 2017 Jul 20;8:1357. doi: 10.3389/fmicb.2017.01357 (PMC5518651; doi:10.3389/fmicb.2017.01357)
Supplement: Supplementary file 1 [file Table1.docx]

Table S1. Primers used in this study

| Gene | Primer sequence (5'-3') | Annealing temperature (℃) | Amplicon size (bp) |
| --- | --- | --- | --- |
| *qnrS1-S3* | F: CACTTTGATGTCGCAGAT | 50 | 471 |
|  | R: CAACACTACCCAGTGCTT |  |  |
| *qnrS2* | F: ATCGCTCCATGGGACTC | 50 | 216 |
|  | R: AACAATACCCAACGCTTC |  |  |
| *qnrA* | F: TTGAGCGGTAAACGAGTGAG | 50 | 823 |
|  | R: GCAACACCGACAGGGAT |  |  |
| *qnrB* | F: TTGAATTCACGGTTTCAGCCCGCTAC | 50 | 875 |
|  | R: TTGGATCCAACGCACATTTGCAGATGTTAT |  |  |
| *qnrC* | F: GAATTATTCCATAAAACG | 55 | 509 |
|  | R: GCTCCCAAAAGTCATC |  |  |
| *aac(6')-Ib-cr* | F: TTGCGATGCTCTATGAGTGG | 51 | 478 |
|  | R: CTCGAATGCCTGGCGTGTTT |  |  |
| *qepA* | F: GCAGGTCCAGCAGCCGGTAG | 55 | 218 |
|  | R: CTCGAATGCCTGGCGTGTTT |  |  |
| *qnrD* | F: CGAGATCAATTTACGGGGAATA | 55 | 582 |
|  | R: AACAAGCTGAAGCGCCTG |  |  |
| *bla*_CTX-M-1_ | F: CAGCGCTTTTGCCGTCTAAG | 52 | 944 |
|  | R: GGCCCATGGTTAAAAAATCACTGC |  |  |
| *bla*_CTX-M-2_ | F: GCATTCGCCGCTCAATGTTA | 50 | 962 |
|  | R: GGTTCGTTGCAAGACAAGAC |  |  |
| *bla*_CTX-M-8_ | F: ACTTCAGCCACACGGATTCA | 50 | 878 |
|  | R:CGAGTACGTCACGACGACTT |  |  |
| *bla*_CTX-M-9_ | F: GTTACAGCCCTTCGGCGATGATTC | 55 | 877 |
|  | R:GCGCATGGTGACAAAGAGAGTGCAA |  |  |
| *bla*_TEM_ | F: TCGGGGAAATGTGCG | 50 | 972 |
|  | R: TGCTTAATCAGTGAGGCACC |  |  |
| *bla*_SHV_ | F: GCCTTTATCGGCCTTCACTCAAG | 55 | 898 |
|  | R: TTAGCGTTGCCAGTGCTCGATCA |  |  |
